# Supplementary material for: Identification of photoperiod-induced specific miRNAs in the adrenal glands of Sunite sheep (Ovis aries)
Source: Front Vet Sci. 2022 Jul 22;9:888207. doi: 10.3389/fvets.2022.888207 (PMC9354845; doi:10.3389/fvets.2022.888207)
Supplement: Supplementary file 1 [file Data_Sheet_1.ZIP › Supplementary file/miRDeep2 file.zip!/miRDeep2 file/Know_Structure/SPLP42A2/oar-mir-329a.pdf]

miRBase precursor : oar-mir-329a
Total read count : 81
oar-miR-329a-5p read count : 13
oar-miR-329a-3p read count : 68
remaining reads : 0

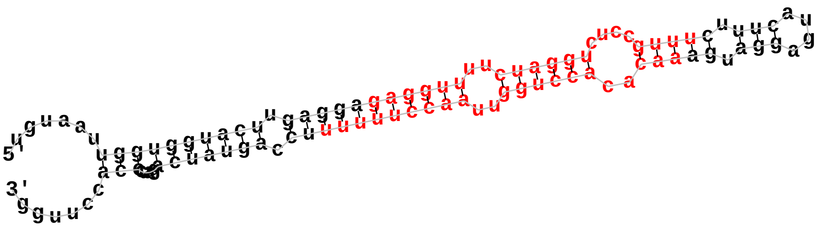

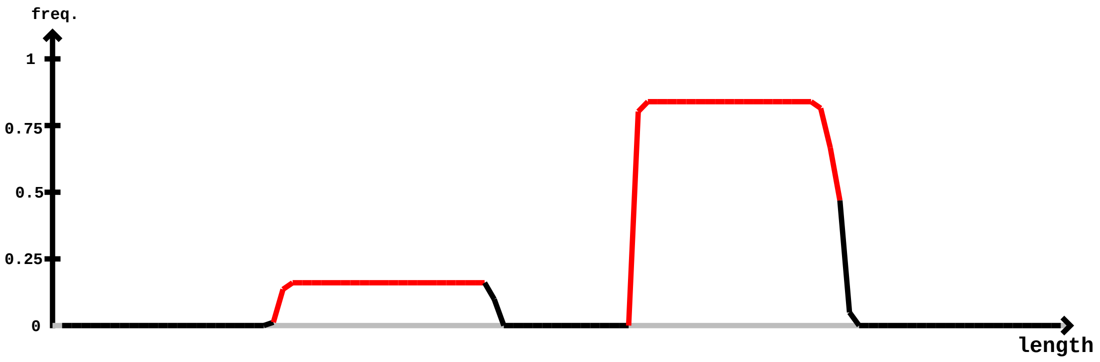


```
                                 oar-miR-329a-5p
                                                                       oar-miR-329a-3p
5'- uguaauuggugguacuugaggagagguuuucuaggucuccguuucuuucaugaggaugaaacacaccugguuaaccuuuuuuccaguaucagaucccaccuugg   -3'   exp
    .......(((((((((.(((((((((((..((((((....(((((.(((....))).)))))..))))))..))))))))))).)))).......))))).... reads   mm   sample
    .....................agagguuuucuaggucuccguuuc........................................................... 1       0    seq
    ......................gagguuuucuaggucuccguuu............................................................ 5       0    seq
    ......................gagguuuucuaggucuccguuuc........................................................... 5       0    seq
    .......................agguuuucuaggucuccguuuc........................................................... 2       0    seq
    ...........................................................aacacaccugguuaaccuu.......................... 2       0    seq
    ...........................................................aacacaccugguuaaccuuu......................... 12      0    seq
    ...........................................................aacacaccugguuaaccuuuu........................ 16      0    seq
    ...........................................................aacacaccugguuaaccuuuuu....................... 33      0    seq
    ...........................................................aacacaccugguuaaccuuuuuuu..................... 2       0    seq
    ............................................................acacaccugguuaaccuuuuu....................... 1       0    seq
    ............................................................acacaccugguuaaccuuuuuu...................... 2       0    seq
```
